# A novel approach for the identification of efficient combination therapies in primary human acute myeloid leukemia specimens

**DOI:** 10.1038/bcj.2017.10

**Published:** 2017-02-17

**Authors:** I Baccelli, J Krosl, G Boucher, I Boivin, V-P Lavallée, J Hébert, S Lemieux, A Marinier, G Sauvageau

**Affiliations:** 1 The Leucegene Project at Institute for Research in Immunology and Cancer, Université de Montréal, Montréal, Québec, Canada; 2 Division of Hematology, Maisonneuve-Rosemont Hospital, Montréal, Québec, Canada; 3 Leukemia Cell Bank of Quebec, Maisonneuve-Rosemont Hospital, Montréal, Québec, Canada; 4 Faculty of Medicine, Department of Medicine, Université de Montréal, Montréal, Québec, Canada; 5 Department of Computer Science and Operations Research, Université de Montréal, Montréal, Québec, Canada; 6 Department of Chemistry, Université de Montréal, Montréal, Québec, Canada

## Abstract

Appropriate culture methods for the interrogation of primary leukemic samples were hitherto lacking and current assays for compound screening are not adapted for large-scale investigation of synergistic combinations. In this study, we report a novel approach that efficiently distills synthetic lethal interactions between small molecules active on primary human acute myeloid leukemia (AML) specimens. In single-dose experiments and under culture conditions preserving leukemia stem cell activity, our strategy considerably reduces the number of tests needed for the identification of promising compound combinations. Initially conducted with a selected library of 5000 small molecules and 20 primary AML specimens, it reveals 5 broad classes of sensitized therapeutic target pathways along with their synergistic patient-specific fingerprints. This novel method opens new avenues for the development of AML personalized therapeutics and may be generalized to other tumor types, for which *in vitro* cancer stem cell cultures have been developed.

## Introduction

Acute myeloid leukemia (AML) is a leading cause of cancer-related death in young adults and represents 100 000 cases per year in G8 countries. Except for acute promyelocytic leukemia, AML treatment has not substantially evolved in the last four decades and remains largely inefficient with a 5-year overall survival of only 26%.^1, 2, 3^ Patients classically receive a ‘7+3' chemotherapeutic induction regimen (7 days of Cytarabine and 3 days of an anthracycline), followed by several courses of consolidation chemotherapy or, if eligible, allogeneic stem cell transplantation.^4, 5^ Regrettably, most remissions remain short-lived as 60–75% of adult patients ⩽60 years old and 85–95% of patients >60 years old still relapse and die from the disease,^6^ most probably due to the outgrowth of leukemic stem cells (LSCs).

Similar to healthy hematopoietic stem cells for the bone marrow tissue, LSCs are able to reconstitute the disease, have a long-term self-renewing capacity and can resist conventional chemotherapeutic treatment.^7, 8, 9, 10, 11, 12^ Despite this great leap forward in the understanding of the cellular biology of the disease 20 years ago, culture methods able to maintain LSC activity of human primary samples *in vitro* were only developed recently, eventually enabling relevant cell-based interrogation of the disease.^13^

With the notable exception of mutations affecting *IDH1*, *IDH2*, *JAK2* and *CSF3R*^14, 15^ (representing roughly 20% of patients), mutations detected in AML are either currently difficult to target (among others: *NPM1*, *DNMT3A, TET2*, *RUNX1*, *TP53*, *ASXL1*, *SRSF2*) or sub-clonal (mutations affecting *KIT*, *FLT3*, *N/KRAS* or *PTPN11*.^16, 17, 18^ There is therefore an urgent need to find novel therapeutic targets that will efficiently kill founder AML LSC clones.

Recent advancements in the development of targeted therapies have highlighted tumor cells' capacity to circumvent the blockade of one particular molecular switch, as is well documented in the cases of *BCR*-*ABL*-positive chronic myeloid leukemia,^19, 20, 21^ non-small-cell lung cancer^22^ or melanoma.^23^ It becomes clear that long-term efficient treatment of cancers will occur through combinations of targeted therapies.^24, 25^ Selecting compounds, which not only target AML founder LSC clones but also synergize together, will have the potential to simultaneously increase treatment efficiencies and reduce their associated side effects in patients.

It is now well accepted that AML represents several distinct entities, with high genetic complexity, not always accurately defined by standard cytogenetic methods. Given this complexity, we predicted that the chemical interrogation of heterogeneous AML primary specimens for novel therapeutic targets would further increase this complexity. We therefore looked for bio-statistical methods, which help associate chemical activities to molecular features of assessed biological samples. Such methods, relying on compound clustering, have been previously reported in yeast, fungus and cancer cell lines but never with primary specimens.^26, 27, 28^

Human cancer cell lines are unfortunately in most cases not able to recapitulate the extent of the complexity of the human disease. Our study is part of the Leucegene initiative in which RNA sequencing of 452 primary AML specimens from various genetic subgroups was performed.^16^ In this particular setting, we are in a unique position to access fully genetically and clinically characterized primary AML samples. We chemically interrogated 20 such specimens with a library of ~5000 compounds, using recently established LSC-maintaining culture conditions,^13^ and developed a novel two-pronged approach to analyze the results of this complex chemo-genomic screen: we first identified subsets of compounds sharing similar activity profiles using a correlation-based clustering method, which we entitled the Compound Correlation Cluster (CCC) method. Subsequently, we used the results of this clustering to assess potential synergistic pairs of relevant compounds in AML.

## Materials and methods

### Human specimens

This study is part of the Leucegene project, approved by the Research Ethics Boards of University of Montréal and Maisonneuve-Rosemont Hospital (Montreal, QC, Canada). All AML samples were collected with an informed consent according to Quebec Leukemia Cell Bank procedures. Mobilized peripheral blood samples were collected from healthy consenting donors according to ethically approved procedures at Maisonneuve-Rosemont Hospital. Human CD34-positive cells were isolated using a CliniMACS Separator (Miltenyi Biotec, Bergisch Gladbach, Germany), according to the manufacturer's instructions.

Sample selection from the primary screen for the synergy studies was based on the following criteria: specimen availability, representation of a maximum of CCC therapeutic classes (see Supplementary Figure 1) and exclusion of hypersensitive specimens (defined by displaying percentages of inhibition in the primary screen >95% for at least three of the five compounds studied).

### CCC determination

Percentage of inhibition data for selective hit compounds were rank-transformed and clustered by minimum spanning tree. Groups of molecules in icicle peaks with *σ*>0.9 were selected and further filtered for elimination of outlier compounds by selection of profiles correlating with *r*>0.9 to the median of the group. The remaining compounds were selected as part of a CCC and are listed in Supplementary Table 1.

### Cell culture

Frozen AML mono-nucleated patients cells were thawed at 37 °C in Iscove's modified Dulbecco's medium containing 20% fetal bovine serum and DNase I (100 μg/ml). Cells were then cultured in a medium designed to support primary AML LSC activity, as previously reported:^13, 16^ Iscove's modified Dulbecco's medium, 15% BIT (bovine serum albumin, insulin, transferrin; StemCell Technologies, Vancouver, British Columbia, Canada), 100 ng/ml stem cell factor, 50 ng/ml FLT3-L, 20 ng/ml, interleukin-3, 20 ng/ml granulocyte colony-stimulating factor (Shenandoah Biotechnology, Warwick, PA, USA), 10^−4^ M β-mercaptoethanol, 500 nM SR1 (Alichem P&C, Monza MB, Italy), 500 nM UM729 (synthesized at the Medicinal Chemistry Core Facility at the Institute for Research in Immunology and Cancer), gentamicin (50 μg/ml) and ciprofloxacin (10 μg/ml).

Human CD34+ cells were cultured as previously described.^29^ Briefly, cells were cultured in HSC expansion media consisting of StemSpan SFEM (StemCell Technologies) supplemented with human 100 ng/ml stem cell factor, 100 ng/ml FMS-like trysine kinase 3 ligand (FLT3LG), 50 ng/ml thrombopoietin (R&D Systems, Minneapolis, MN, USA) and 10 μg/ml low-density lipoproteins (StemCell Technologies).

### Cell viability assays

Primary cells were seeded in 384-well plates at a density of 5000 cells in 50 μl per well for AML specimens and at a density of 2000 cells in 50 μl per well for normal controls. In the primary screen, compounds were added to seeded cells at concentrations varying from 1 μM to ~15 μM. Each compound in Supplementary Table 2 was tested in a single well (see Compounds section of Materials and methods, and Supplementary Figure 2). In the dose–response combinatorial assays with CCC representative compounds, each molecule was added to seeded cells at five different doses (0, 100 nM, 500 nM, and 1 and 5 μM) and each dilution was tested in quadruplicates.

In all experiments, wells treated with 0.1% dimethylsulfoxide (DMSO) without additional compound were used as negative controls. In the primary screen, each test plate included positive controls Cytarabine and Daunorubicin tested at 1 and 0.05 μM concentrations. In dose–response assays, positive control wells were treated with 6-thioguanine. In all experiments, cell viability was evaluated after 6 days of culture. In the primary screen viable fluorescent calcein-positive cells (Thermo Fisher Scientific, Waltham, MA, USA) were counted on five areas corresponding to 12.5% of the well surface (using the high-content-imaging device Operetta (Perkin Elmer, Waltham, MA, USA; see Supplementary Figure 2). In all other experiments, cell viability was assessed with the CellTiterGlo assay (Promega, Madison, WI, USA). Percentages of inhibition were calculated as follows: 100−(100 × (average signal in compound-treated wells)/(average signal in DMSO-treated wells)).

In dose–response experiments, EC50 values (corresponding to the concentration of compound required to reach 50% of inhibition) were calculated using ActivityBase SARview Suite (IDBS, London, UK) and GraphPad Prism 4.03 (La Jolla, CA, USA).

In the CCC combinatorial screen, synergism between drugs was evaluated using the PRECISE software, according to the method described in Dietlein *et al.*, 2015.

### Compounds

All powders were dissolved in DMSO and diluted in culture medium immediately before use. Final DMSO concentration in all conditions was 0.1%. The suppliers for each compound tested are listed in Supplementary Table 2. Off-patent compounds and epigenetic response modifiers were tested at 2.5 μM, commercially available and Institute for Research in Immunology and Cancer proprietary compounds at 5 ng ml^−1^ (equivalent of around 15 μM) and kinase inhibitors at 1 μM.

### Statistics

*P*-values and Pearson's correlation coefficients were calculated using GraphPad Prism 4.03 (La Jolla, CA, USA). Hierarchical clustering of patients according to their CCC median profiles was carried out using MeV 4.8. Correlation of compounds into CCCs is described in the Methodology for CCC determination section of Materials and Methods. Enrichment for mutations, genetic group or French American British statuses were probed for significance using a Fisher's exact test with a Bonferroni correction. Analysis of differential gene expression was performed using the Wilcoxon's rank-sum test and the false discovery rate method was applied for global gene analysis as previously described.^16^ Significance levels were set at false discovery rate *q*-value<0.05.

## Results

### Results of a 20-AML specimen chemical screen

**Figure 1:**
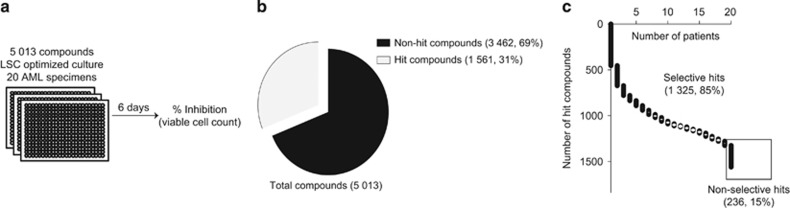
Primary screen overview. (**a**) Overview of the primary screen workflow. (**b**) Pie-chart representation of hit compounds (drugs achieving >50% inhibition compared with DMSO-treated controls in at least one sample of the cohort) versus non-hit compounds. (**c**) Frequencies of selective (effective in 1–19 samples) versus non-selective (effective in all 20 specimens) hits. AML, acute myeloid leukemia; LSC, leukemic stem cells.

We interrogated 20 genetically diverse AML patient specimens with a library of 5013 small molecules, highly enriched with off-patent compounds (Figure 1a and Supplementary Figure 2a, list of compounds, raw data, and patient clinical and mutational information in Supplementary Table 2). Patient samples included in this assay displayed very heterogeneous proliferation patterns (Supplementary Figure 2b), highlighting the fact that each specimen is unique, and that this screen represents in fact 20 different screens.

Hit compounds (drugs achieving >50% inhibition compared with DMSO-treated controls in at least one sample of the cohort) were highly frequent (31%, 1561/5013, Figure 1b), likely due to the large number of individual specimens tested and to a strong bias towards biologically active compounds, most of which represent validated drugs with established cellular activity. Within hit compounds, 15% drugs (236/1561) were non-selective (effective in all 20 specimens), whereas 85% (1325/1561) were selective (effective in a subset of samples, Figure 1c).

**Figure 2:**
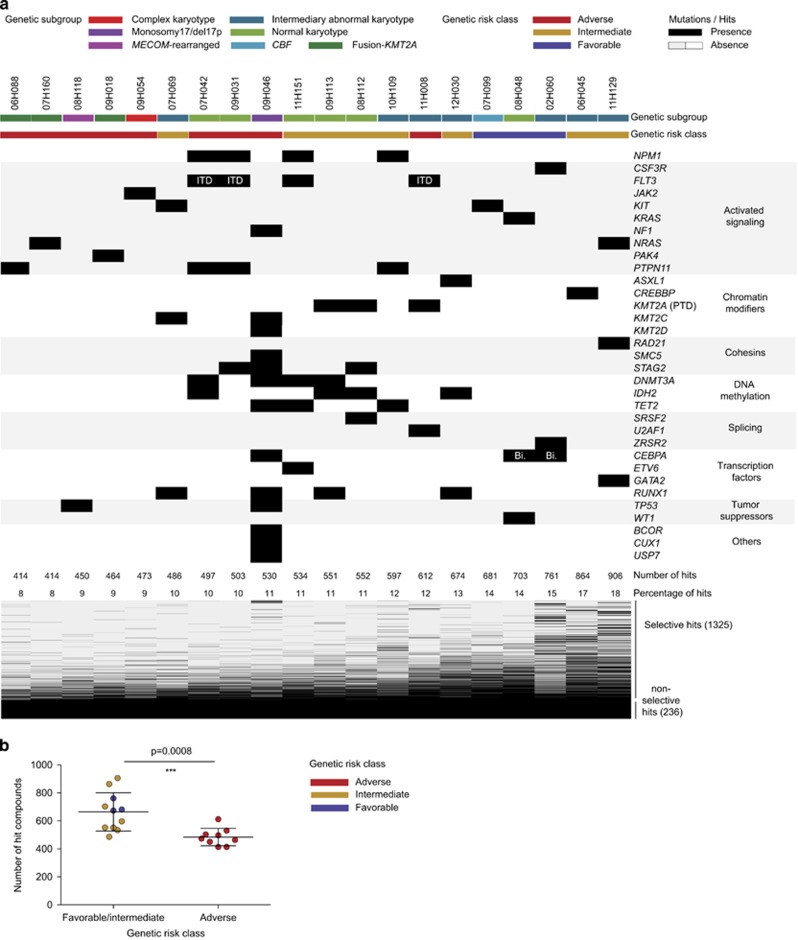
Primary screen results. (**a**) Overview of the genetic diversity of the 20 AML specimens included in the primary screen and the number and frequencies of hits per patient. (**b**) Number of hits with regards to genetic risk classes. *P*-values were assessed by Mann–Whitney test and data are presented as mean±s.d. Bi, bi-allelic; ITD, internal tandem duplication; PTD, partial tandem duplication.

The hit rate per patient varied from 8% (414/5013) to 18% (906/5013) with a median rate per patient of 11% (543/5013, Figure 2a). Patients with the five lowest hit rates (<10%) were all of adverse genetic-risk class: *KMT2A*-fusion samples (3/3, 06H088, 07H160 and 09H018), complex karyotype (09H054), *MECOM* rearranged and *TP53* mutated (08H118; Figure 2a). Overall, AML patients with adverse genetic risk had significantly lower hit rates than patients with intermediate and favorable genetic risk (Figure 2b).

### Identification of CCCs

**Figure 3:**
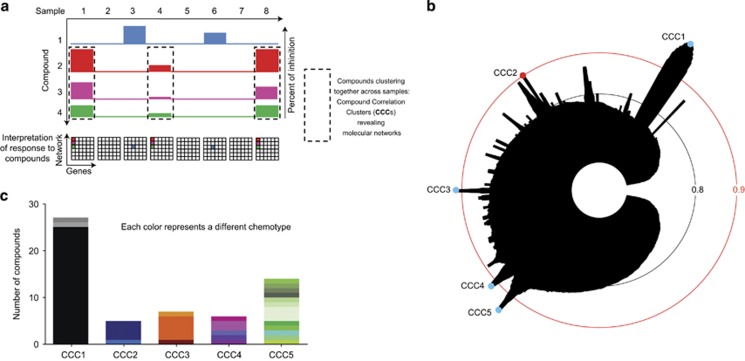
Identification of CCCs. (**a**) Scheme summarizing the concept of CCCs. (**b**) Icicle representation of selective hit compounds of the primary screen highlighting five peaks of strongly correlating compounds (CCCs) with *σ*⩾0.9. (**c**) Each CCC is composed of compounds belonging to several chemotypes (series of chemical entities that share a similar scaffold), which are specific to a given CCC. CCC, Compound Correlation Cluster.

We hypothesized that chemical interrogation of patient samples is able to integrate the complex molecular networks that are essential to tumor cell survival and/or proliferation. More specifically, we predicted that selective compounds inducing similar patterns of inhibition across samples are revealing the presence of therapeutic target pathways specifically sensitized in a subset of patient samples. The term CCC was coined for such groups of drugs (Figure 3a).

**Figure 4:**
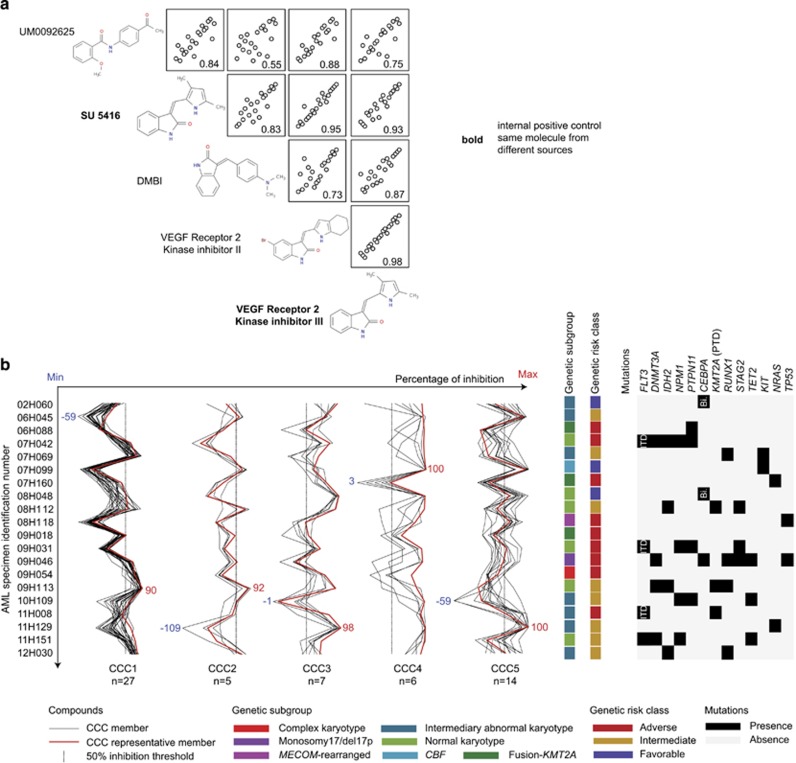
CCC inhibitory patterns. (**a**) Example of correlation matrixes between compounds belonging to CCC2. (**b**) Inhibitory patterns of the five CCCs in the primary screen cohort and corresponding genetic sample main characteristics (only genes mutated in ⩾2 samples are shown). CCC representative compounds are highlighted in red. AML, acute myeloid leukemia; Bi, bi-allelic; CCC, Compound Correlation Cluster; ITD, internal tandem duplication; Max: maximum; Min, minimum; PTD, partial tandem duplication; VEGF, vascular endothelial growth factor.

To test this hypothesis, we clustered rank-transformed inhibitory data of our selective hit compounds using single-linkage hierarchical clustering. The resulting clusters were investigated using an icicle representation and revealed the presence of five different CCCs (CCC1–5, *σ*>0.9 Figure 3b). After elimination of outlier profiles (*r*<0.9 with median profile of the group), we obtained a list of molecules belonging to several different CCC-specific chemotypes (series of chemical entities that share a similar scaffold, Figure 3c), inducing highly correlating response patterns across AML specimens (Figures 4a and b). The detailed list of compounds and chemotypes of CCC1–5 is provided in Supplementary Table 1. Molecular structures and correlation matrices of CCC2 members are shown in Figure 4a. As an internal positive control, the same molecule purchased from two different suppliers did cluster in CCC2 with *σ*=0.93 (in bold in Figure 4a). The response patterns of the five CCCs shown in Figure 4b were distinct from one another. One representative compound per CCC (highlighted in red in Figure 4b and in Supplementary Table 1) was selected according to its level of activity in patient cells, the correlation of its sensitivity patterns to the median profile of the cluster and to its commercial availability.

### CCCs targets are yet to be identified

Although a large number of patients would be required to make a definitive statement, CCCs did not appear to associate with a particular genetic subgroup, French–American–British subtype or with the presence of specific mutations (Figure 4b and Supplementary Figure 3a).

Comparison of patient transcriptomic profiles depending on their sensitivities to CCCs did not reveal any differentially expressed gene between sensitive and resistant patients (not shown). Furthermore, a large proportion of the CCC representative compounds' putative targets are not expressed in the samples tested (non-exhaustive list in Supplementary Table 5). Taken together, these results suggest that most of the CCCs we identified might be acting via yet unidentified target(s) in AML cells.

### Synergistic fingerprinting of CCCs identifies recurrent synergistic combinations

Starting with a set of approximately 10^5^ data points (5000 compounds interrogating 20 AML samples with next-generation sequencing information and clinical annotations), our approach allowed us to extract five CCC-representative drugs displaying distinct response profiles. Owing to the low number of compounds thus selected, we were able to carry out patient sample cell-based synergistic studies.

**Figure 5:**
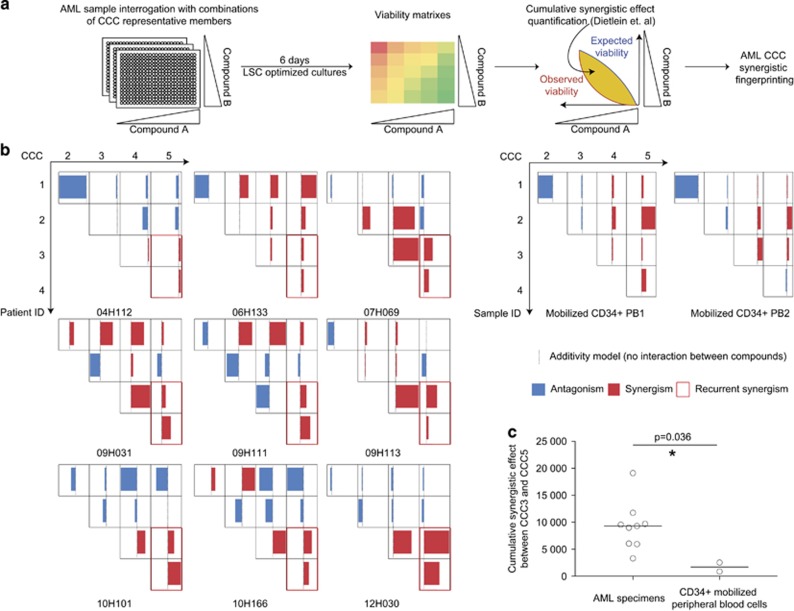
CCC synergistic fingerprints. (**a**) Synergy screen workflow overview. (**b**) CCC synergistic fingerprints of AML samples (left panel) and normal CD34-positive, mobilized peripheral blood samples (right panel). Cumulative synergistic effects were obtained as described in Dietlein *et al.*, 2015. Red indicates synergism (positive cumulative effect) and blue antagonism (negative cumulative effects). Recurrent synergistic interactions are highlighted with thicker red boxes. Raw data are available in Supplementary Table 6. (**c**) Cumulative synergistic effects of CCC3 and CCC5 representative compound combinations in AML specimens (*n*=9) and in normal controls (*n*=2). Significance was probed by Mann–Whitney test. AML, acute myeloid leukemia; CCC, Compound Correlation Cluster; LSC, leukemia stem cell, PB, peripheral blood.

First, hierarchical clustering of patients based on their median CCC profiles potentially identifies 10 different AML therapeutic classes, the molecular target(s) of which remain to be identified (Supplementary Figure 1). These therapeutic classes do not reflect known genetic risk classes, mutational status or genetic subgroups of AML (Supplementary Figures 1 and 3b). Second, nine AML specimens were selected for synergy analysis, four of which belonging to the primary screen (highlighted in red in Supplementary Figure 1). Third, dose–response combinatorial assays of CCC representative drugs were carried out in order to determine viability matrices, allowing for the quantification of cumulative synergistic effects as described in Dietlein *et al.*,^30^ 2015 and subsequent CCC synergistic fingerprinting of AML samples, as well as normal CD34-positive mobilized peripheral blood cell controls (Figures 5a and b).

Analysis of single compound dose responses confirmed our primary screen data (*r*=−0.8, Supplementary Figure 4a). All combinations of CCC compounds tested yielded a deviation from the additivity model, the cumulative synergistic effects varying from −53 725 (strong antagonism) to 38 361 (strong synergism) in AML samples and from −30 863 to 13 424 in normal cells (Figure 5b and Supplementary Table 6). AML specimens had on average a greater deviation from the additivity model than normal controls (median 10 007 versus 3 762, *P*=0.0005, Supplementary Figure 4b). Most of the interactions between CCC compounds appeared sample specific: for instance, CCC1 and CCC4 strongly synergized in patient 09H111, whereas they strongly antagonized in patient 10H101, suggesting that the nature of some of these interactions is context dependent. However, one pair of CCC representative compounds synergized in the vast majority of specimens tested (CCC3–CCC4, 8/9 specimens, Figure 5b and examples in Supplementary Figure 4c) and two pairs synergized in all tested specimens (CCC3–CCC5 and CCC4–CCC5, highlighted in bold red in Figure 5b). Moreover, the cumulative synergistic effects of CCC3–CCC5 were significantly higher in AML specimens than in normal CD34-positive control cells (median 9 300 versus 1 703, *P*=0.036, Figure 5c).

### Different members of a CCC induce similar responses and synergy patterns in AML

**Figure 6:**
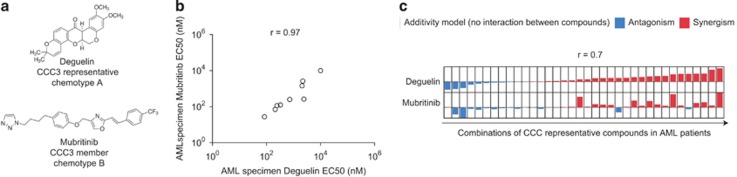
Inhibitory patterns and synergistic fingerprints of two distinct compounds of a same CCC. (**a**) Chemical structures of two members of CCC3: Deguelin and Mubritinib. (**b**) Correlation between EC50 values of Deguelin and Mubritinib in AML cells. (**c**) Comparison of the synergistic patterns of Deguelin and Mubritinib. AML, acute myeloid leukemia; CCC, Compound Correlation Cluster.

In order to test the extent of the similarity of action of compounds within a given CCC, synergistic fingerprints of two chemically different members of CCC3 were assessed: Deguelin and Mubritinib (Figure 6a). Deguelin is a natural insecticide derived from leguminous plants, classified as a rotenoid of the flavonoid family.^31^ It has shown anti-cancer activity both *in vitro* and *in vivo* in diverse cancers.^32, 33, 34, 35, 36, 37, 38, 39, 40^ Deguelin is thought to inhibit various molecular pathways, such as, among others, the phosphoinositide 3-kinase/protein kinase B (AKT)/and mitogen-activated protein kinases/ERK pathways^32, 33, 34, 35, 36, 37^ or the nuclear factor κ-light-chain enhancer of activated B-cell pathway.^38, 39, 40^ Mubritinib, on the other hand, is a receptor tyrosine kinase ERBB2 inhibitor^41^ having an extended acyclic structure unrelated to the flavonoid family. The two CCC3 members display highly correlating EC50 values (*r*=0.95, Figure 6b) in AML cells. Moreover, we observed a noticeable similarity (*r*=0.7) between the synergistic/antagonistic patterns of both drugs when used in combinations with other CCC representative compounds in patient cells (Figure 6c). These data indicate that both CCC3 members, which belong to distinct chemotypes and have been reported to have different mechanisms of action, are nevertheless revealing the same molecular target pathway(s) in AML, as initially hypothesized.

## Discussion

As synergistic studies are highly cell- and compound-consuming, they cannot be carried out on a large-scale basis with primary human specimens. Here we used the concept of compound clustering previously developed in human cancer cell line screens^26^ to extract relevant clusters of compounds of interest from our compound inhibitory data, enabling synthetic lethality screening in primary leukemia specimens. All combinations of CCC representative compounds led to either antagonistic or synergistic effects in patient cells (Figure 5b and Supplementary Table 6), suggesting that target pathway(s) are specific to each cluster. Moreover, in a small cohort of AML patients, we detected the presence of two recurrent synergistic CCC interactions, one of them being significantly higher than in normal CD34-positive blood cells, suggesting that despite the genetic complexity of AML, some therapeutic targets may be synchronous, and that only a handful of combinatorial treatments might be sufficient to treat the majority of patients without major toxicity.

The fact that Deguelin and Mubritinib induce the same inhibitory patterns (Figure 6b), as well as similar synergistic patterns (Figure 6c) in leukemic cells, when combined with compounds of other CCCs, strongly suggests that CCCs are able to group structurally unrelated molecules, which, in the specific context of primary AML cells, identify the same target pathway(s).

As illustrated by the poor overall survival rate in AML, current classifications of the disease do not suffice to assign correct treatments to patients. The paradigm according to which the presence of one mutation or one genetic alteration warrants the administration of one corresponding drug appears to be less straight forward than previously believed. Accordingly, response patterns to CCCs did not correlate with specific mutations, gene expression patterns or genetic subgroup, highlighting dissociation between our current knowledge of the disease and the CCC-uncovered therapeutic targets.

Regardless of the fact that specific pathways targeted by CCCs remain to be identified, the CCC approach provides a solid starting point for the development of novel therapeutic strategies in AML. First, by integrating the data from a large number of specimens (genetic diversity) and compounds (structural diversity), CCCs are able to reveal sensitized pathways even when the frequency of the event is low. Second, already clinically approved compounds (in the context of other diseases) emerging in a CCC might benefit from repositioning protocols, which allow for proof-of-concept clinical trials to be carried out before spending years of research investment in drug discovery. In addition, this can warrant patients access to effective compounds more rapidly than through classical routes, while giving precious time to the generation of novel more effective molecules. Third, because CCCs gather drugs from different chemotypes and with different reported mechanisms of actions, target identification of such molecules might be greatly accelerated by intersection (or elimination) of the different associated candidates. Last, CCC identification is achievable with single-dose screening strategies.

Although a few interesting hits from the approximately 5000-molecule collection of this proof-of-concept study can already be exploited, the strength of this approach will be greatly enhanced in the future by the exploration of more diversified compound collections and patient cohorts. Such a study would increase our chances of finding new efficient combination therapies with a special interest for poor prognostic AML patients.

Finally, as this study can easily be generalized to other tumor types for which cancer stem cell relevant culture methods exist, we believe that it will be used as a starting point for the search of novel therapeutic combination therapies in other cancer entities.
