# Supplementary material for: A novel approach for the identification of efficient combination therapies in primary human acute myeloid leukemia specimens
Source: Blood Cancer J. 2017 Feb 17;7(2):e529–. doi: 10.1038/bcj.2017.10 (PMC5386329; doi:10.1038/bcj.2017.10)
Supplement: Supplementary Information [file bcj201710x1.pdf]

Supplementary Information for

A Novel Approach For The Identification of Efficient Combination Therapies In Primary Human Acute Myeloid Leukemia Specimens

Irène Baccelli, Jana Krosl, Geneviève Boucher, Isabel Boivin, Vincent-Philippe Lavallée, Josée Hébert, Sébastien Lemieux, Anne Marinier and Guy Sauvageau
Correspondence to: guy.sauvageau@umontreal.ca and anne.marinier@umontreal.ca

This PDF file includes:

Supplementary Figures 1-4
Supplementary Tables 1 and 3-6

Other Supplementary Materials for this manuscript includes the following:

Supplementary Table 2 in xlsx format

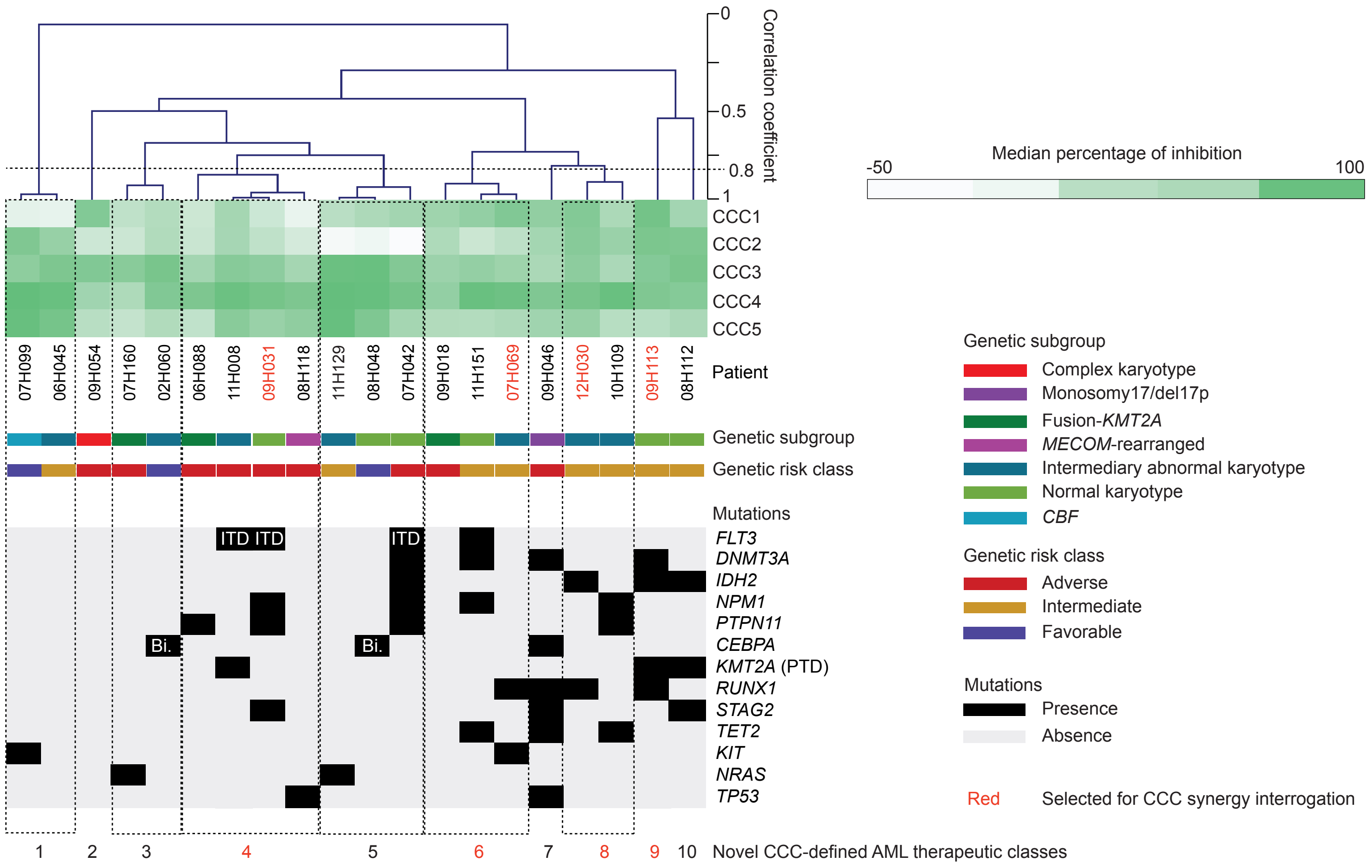

Supplementary Figure 1
Hierarchical clustering of patient specimens based on their CCC profiles.
Hierarchical clustering of patient specimens and their corresponding main genetic characteristics (only genes mutated in ≥2 samples are shown) according to their median CCC inhibitory patterns, leading to the identification of ten novel classes of samples (threshold r=0.8). Shown in red are patient samples selected for experiments exposed in **Fig. 3a-d**.
Abbreviations: AML: Acute Myeloid Leukemia; Bi: bi-allelic; CCC: Compound Correlation Cluster; ITD: internal tandem duplication; PTD: partial tandem duplication.

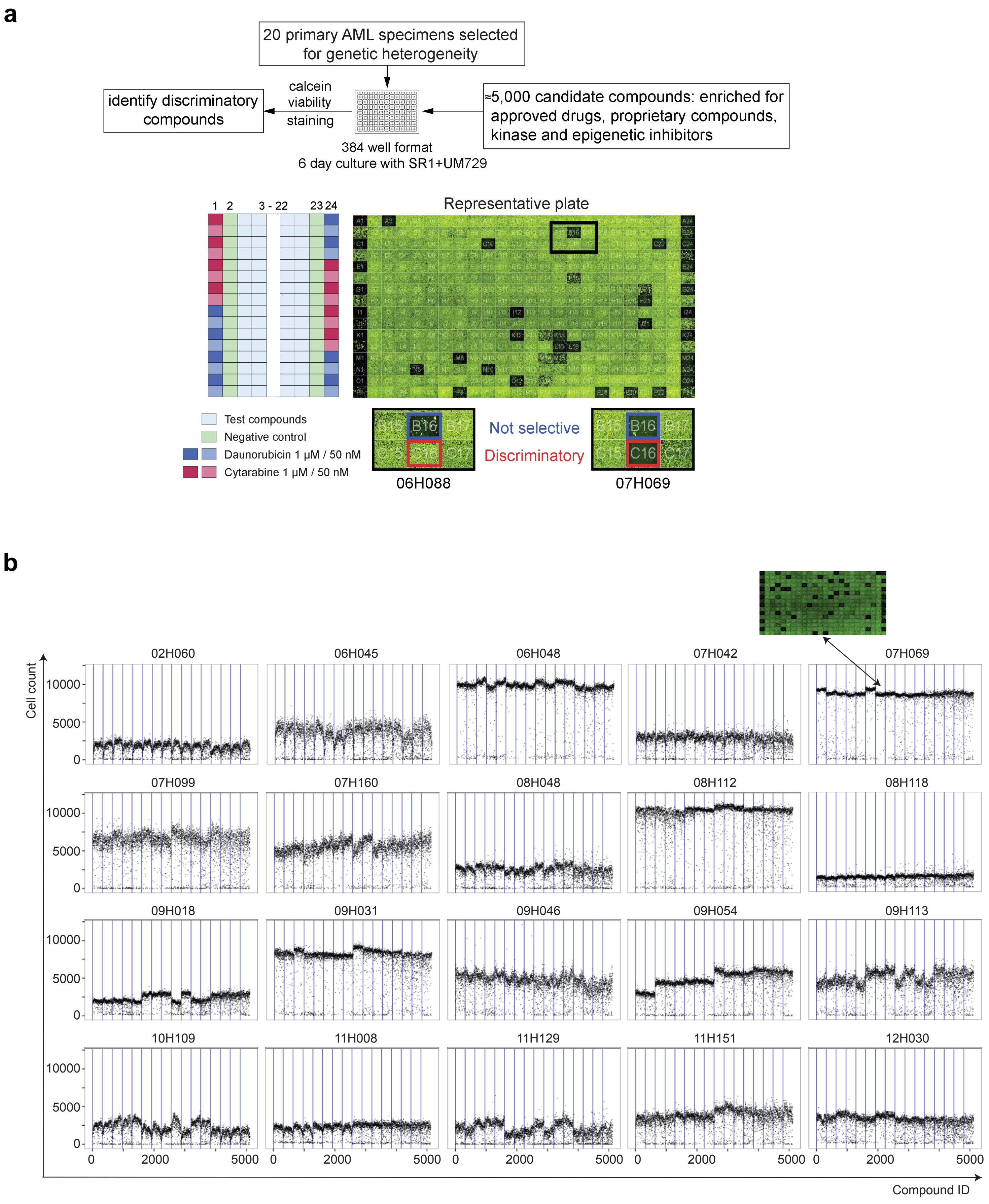

Supplementary Figure 2

Primary screen set-up.

(**a**) Detailed workflow and set-up of the primary screen, showing an example of a Calcein stained 384-well plate. (**b**) Overview of the diversity of cell counts per patient and per compound. Each lane corresponds to a different 384-well plate, and dots represent viable cell counts in each test well. Abbreviations: AML: Acute myeloid leukemia; DNA: deoxyribonucleic acid; SR1: StemRegenin.

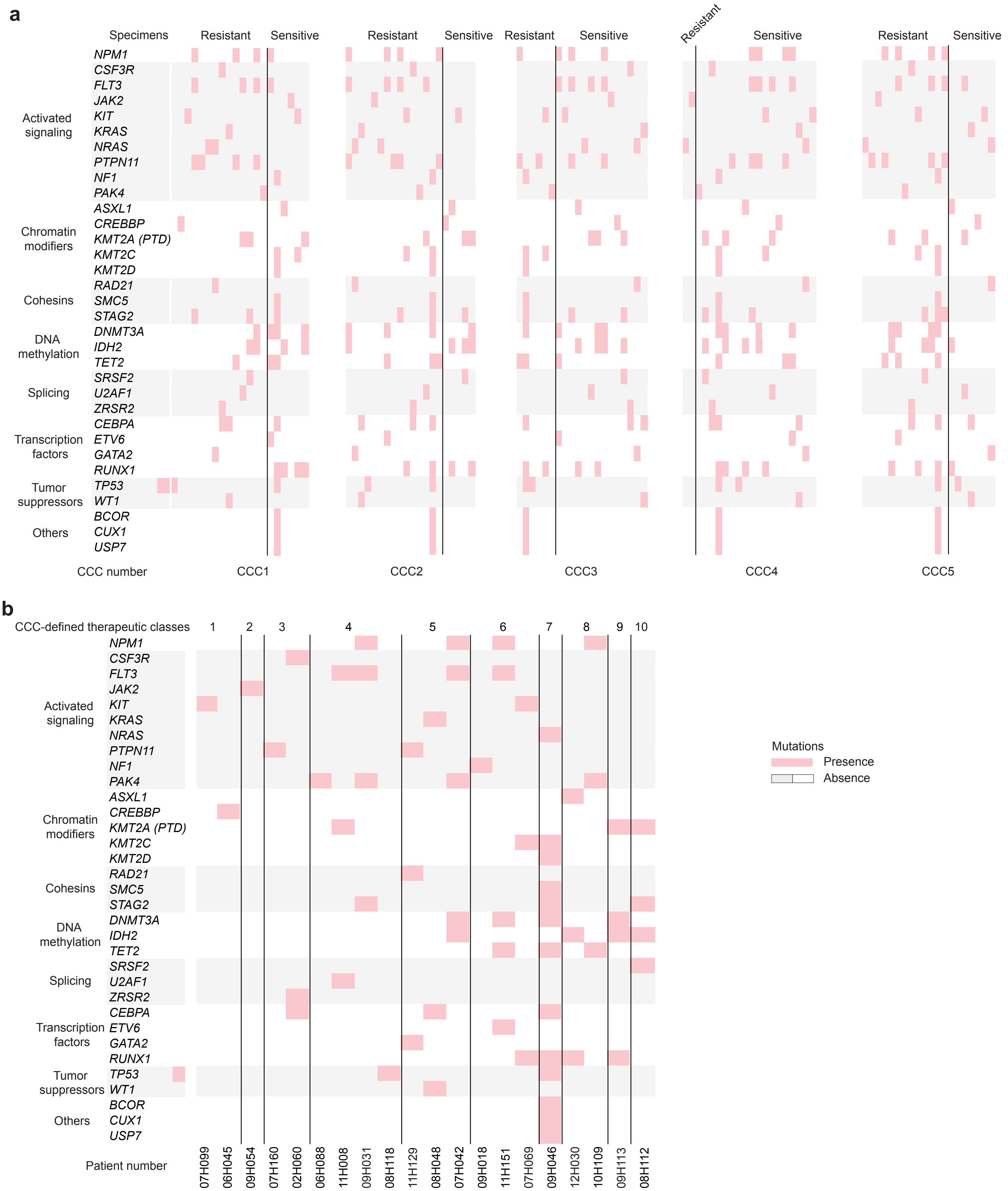

Supplementary Figure 3

Mutations and response to compounds.

Mutations detected in AML samples according to (**a**) their sensitivity or resistance to CCC compounds (the median sensitivity profile of each cluster was used) or (**b**) to their CCC-defined therapeutic classes as shown in **Sup. Figure 1**. Abbreviations: CCC: Compound Correlation Cluster; PTD: partial tandem duplication.

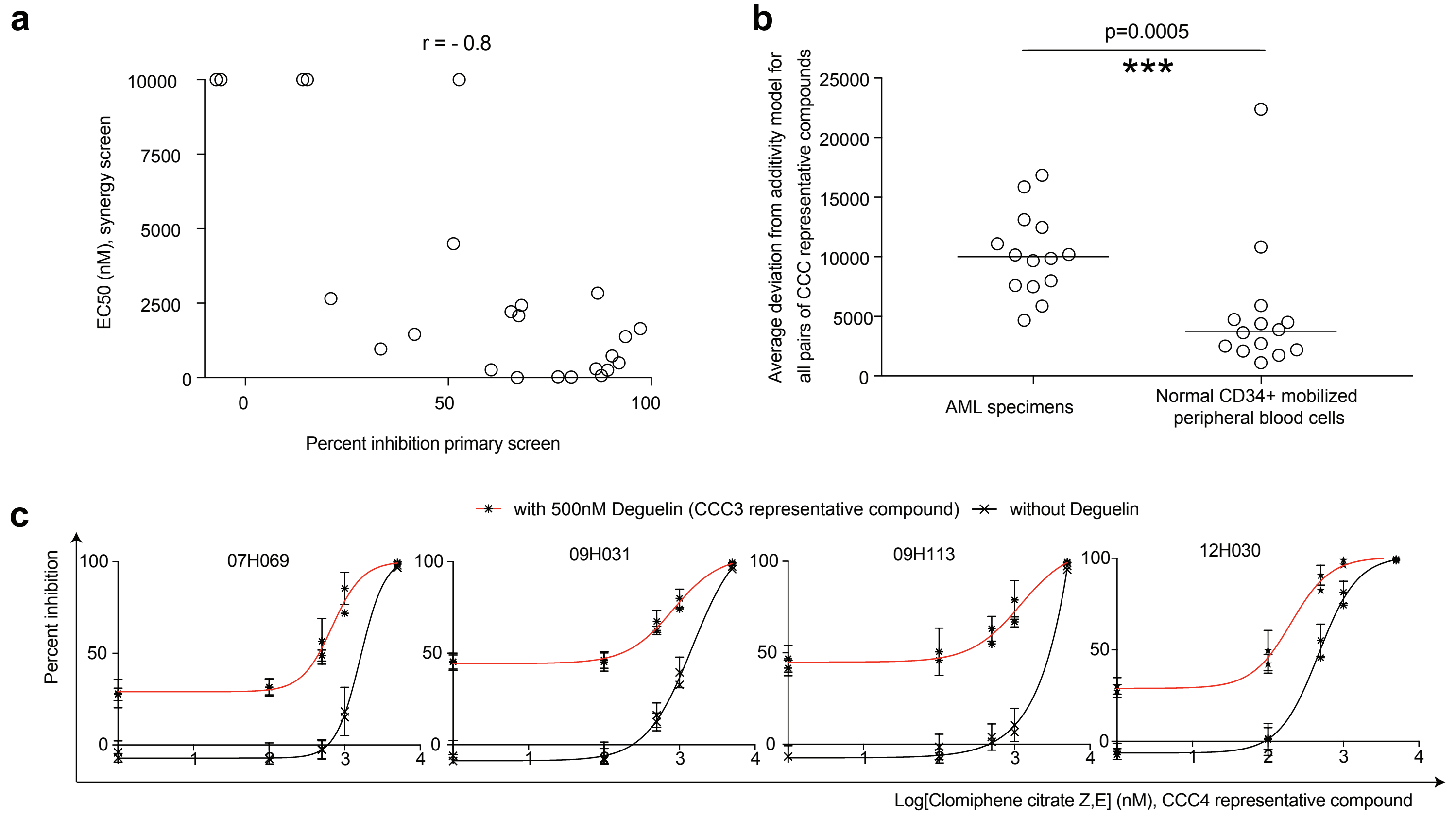

Supplementary Figure 4

Synergistic screen complementary data.

(**a**) Correlation between percentage of inhibition obtained in the primary screen (one concentration, one well per compounds) and EC50 values obtained in the synergistic screen (dose response assays, 5 dilutions, 4 wells per dilution). (**b**) Average deviation from the additivity model for all CCC interactions tested (including Mubritinib) in AML specimens (n=9) and normal CD34 positive mobilized peripheral blood cells (n=2). Differences were probed for significance by Wilcoxon test. (**c**) Examples of synergy between Deguelin (CCC3, 500nM) and CCC4 representative Clomiphene citrate in inhibiting patient cell proliferation/survival determined by dose response assays.

Abbreviation: CCC: Compound Correlation Cluster.

CCC number	Compound number	Compound name	Chemotype
CCC001	UM0118209	Dexamethasone acetate	1
	UM0118277	Fluorometholone	1
	UM0118279	Flumethasone	1
	UM0118385	Prednisolone	1
	UM0118403	Triamcinolone	1
	UM0118534	Methylprednisolone, 6-alpha	1
	UM0118554	Fludrocortisone acetate	1
	UM0118601	Betamethasone	1
	UM0118717	Budesonide	1
	UM0118790	Mometasone furoate	1
	UM0118822	Beclomethasone dipropionate	1
	UM0118888	Fluocinonide	1
	UM0118891	Halcinonide	1
	UM0118916	Clocortolone pivalate	1
	UM0118920	Diflorasone Diacetate	1
	UM0119040	Fluticasone propionate	1
	UM0119106	Rimexolone	1
	UM0119595	Beclomethasone	1
	UM0120482	6alpha-METHYLPREDNISOLONE ACETATE	1
	UM0120829	KARANJIN	2
	UM0121251	NAPROXOL	3
	UM0121423	AMCINONIDE	1
	UM0121425	DEXAMETHASONE	1
	UM0121443	FLUDROCORTISONE ACETATE	1
	UM0121454	TRIAMCINOLONE ACETONIDE	1
	UM0121463	BETAMETHASONE VALERATE	1
	UM0121473	PREDNISOLONE ACETATE	1
CCC002	UM0092625	No name	1
	UM0120215	SU 5416	2
	UM0127844	DMBI	2
	UM0127939	VEGF Receptor 2 Kinase Inhibitor II	2
	UM0127940	VEGF Receptor 2 Kinase Inhibitor III	2
CCC003	UM0102285	No name	1
	UM0119166	DEGUELIN	2
	UM0120796	MUNDULONE	2
	UM0120884	alpha-TOXICAROL	2
	UM0120957	MUNDOSERONE	2
	UM0121114	beta-TOXICAROL	2
	UM0127955	Mubritinib	3
CCC004	UM0092226	No name	1
	UM0099366	No name	2
	UM0103206	No name	3
	UM0118242	Econazole nitrate	4
	UM0118646	Clomiphene citrate (Z,E)	5
	UM0121481	MICONAZOLE NITRATE	4
CCC005	UM0092562	No name	1
	UM0094107	No name	2
	UM0098818	No name	3
	UM0104722	No name	4
	UM0105815	No name	5
	UM0118177	Proadifen hydrochloride	6
	UM0118389	Prochlorperazine dimaleate	7
	UM0118582	Clemastine fumarate	6
	UM0118868	Pramoxine hydrochloride	8
	UM0119025	Deptropine citrate	9
	UM0119089	Piperidolate hydrochloride	10, enantiomer
	UM0121007	DERRUBONE	11
	UM0121477	HYDROXYZINE PAMOATE	12
	UM0125497	No name	6

Supplementary Table 1
List of compounds and corresponding chemotypes in Compound Correlation Clusters (CCCs). Representative compounds are shown in red.

Supplementary Table 3
Patient clinical data.

Patient number	Sex	Age	White Blood Cell count (x10^9/L)	French American British subtype	World Health Organization 2008 classification
02H060	M	84	99.2	M1	AML without maturation
04H112	F	64	361.2	M1	AML without maturation
06H045	F	30	16.6	M2	AML with maturation
06H088	M	28	26.4	M1	AML without maturation
06H133	F	45	11.9	M0	AML with myelodysplasia-related changes
07H042	M	23	69	not classifiable	Acute myeloid leukaemia, NOS
07H069	M	55	80	not classifiable	Acute myeloid leukaemia, NOS
07H099	F	58	61.5	not classifiable	AML with inv(16)(p13.1q22) or t(16;16)(p13.1;q22); CBFB-MYH11
07H160	F	67	74.6	M1	AML with myelodysplasia-related changes
08H048	M	45	127.7	M1	AML without maturation
08H112	M	52	28.3	not classifiable	AML with myelodysplasia-related changes
08H118	F	73	45.9	M0	AML with myelodysplasia-related changes
09H018	M	56	101.8	M0	AML with myelodysplasia-related changes
09H031	F	54	48.9	M1	AML without maturation
09H046	M	37	52	not classifiable	Therapy-related myeloid neoplasms
09H054	F	54	80.3	not classifiable	AML with myelodysplasia-related changes
09H111	F	59	46.8	M5	Acute monoblastic and monocytic leukaemia
09H113	M	56	68.7	M1	AML without maturation
10H101	F	49	24.5	M2	AML with myelodysplasia-related changes
10H109	F	65	318	M1	AML without maturation
10H166	M	63	226.2	M4	Acute myeloid leukaemia, NOS
11H008	M	76	86.2	not classifiable	AML with myelodysplasia-related changes
11H129	M	62	322.5	M1	AML without maturation
11H151	M	61	53.3	M1	AML without maturation
12H030	M	78	144.8	M0	AML with minimal differentiation

Patient number	Karyotype	Genetic subgroup	Genetic risk class*
02H060	45,XY,-21[3]/46,XY[22]	intermediate abnormal karyotype	favorable
04H112	46,XX[21]	normal karyotype	adverse
06H045	46,XX,4~46dmin[22]	intermediate abnormal karyotype	intermediate
06H088	46,XY,t(6;11)(q27;q23)[20]	*KMT2A* fusion	adverse
06H133	46,XX[20]	normal karyotype	adverse
07H042	46,XY[20]	normal karyotype	adverse
07H069	46,XY,inv(7)(q22q36)[20]	intermediate abnormal karyotype	intermediate
07H099	46,XX,inv(16)(p13.1q22)[20]	core binding factor rearrangement	favorable
07H160	45,XX,-7[19]/46,XX[1]_MLL-MLLT10 confirmed by RNA sequencing	*KMT2A* fusion	adverse
08H048	46,XY[21]	normal karyotype	favorable
08H112	46,XY[20]	normal karyotype	intermediate
08H118	43~45,X,-X,t(3;16;6)(q26.2;q22;q25),del(5)(q15q33),add(7)(q22),-14,-17,-17,-20,-22,+1~4mar[cp20]	*MECOM* rearrangement	adverse
09H018	46,XY,del(1)(p13),add(1)(q32),add(11)(q25),t(11;19)(q23;p13.1),t(12;15)(p13;q25),add(17)(q25)[19]/46,XY[1]	*KMT2A* fusion	adverse
09H031	46,XX[20]	normal karyotype	adverse
09H046	45,XY,add(16)(p13.1),-17[20]	monosomy17/del17p	adverse
09H054	46~49,XX,add(5)(q3?5),-6,+8,+9,der(11)t(11;12)(q21;q?15),-12,-14,-16,-18,-20,-21,+1~8mar,2min[cp22]	complex karyotype	adverse
09H111	46,XX[21]	normal karyotype	intermediate
09H113	46,XY[22]	normal karyotype	intermediate
10H101	46,XX[22]	normal karyotype	adverse
10H109	45,XX,der(7)t(7;18)(p12;q12),-18[17]/46,XX[3]	intermediate abnormal karyotype	intermediate
10H166	46,XY[20]	normal karyotype	adverse
11H008	48,XY,+13,+15[3]/46,XY[14]	intermediate abnormal karyotype	adverse
11H129	47,XY,+10[19]/46,XY[1]	intermediate abnormal karyotype	intermediate
11H151	46,XY[21]	normal karyotype	intermediate
12H030	47,XY,+13[3]/46,XY[30]	intermediate abnormal karyotype	intermediate

*based on cytogenetics combined with biallelic CEBPA or FLT3-ITD mutational statuses

Patient number	Gene	Mutation	Position	NM number	Method
02H060	*CEBPA*	Y62X	chr19:33792778	NM_001285829	casava
02H060	*CEBPA*	G96FsX	chr19:33793032	NM_004364	casava
02H060	*CSF3R*	T618I	chr1:36933434	NM_000760	casava
02H060	*ZRSR2*	R427FsX	chrX:15841195	NM_005089	casava
06H045	*CREBBP*	R768X	chr16:3823913	NM_004380	casava
06H088	*PTPN11*	A72V	chr12:112888199	NM_002834	casava
07H042	*DNMT3A*	C586Y	chr2:25467118	NM_022552	casava
07H042	*FLT3*	Q580FsX, ITD	chr13:28608318	NM_004119	kmer
07H042	*IDH2*	R140Q	chr15:90631934	NM_002168	casava
07H042	*NPM1*	L258FsX	chr5:170837544	NM_002520	casava
07H042	*NPM1*	W259FsX	chr5:170837548	NM_002520	kmer
07H042	*PTPN11*	T73I	chr12:112888202	NM_002834	kmer
07H069	*KIT*	N818K	chr4:55599340	NM_001093772	kmer
07H069	*KMT2C*	E4441Q	chr7:151845691	NM_170606	casava
07H069	*RUNX1*	R320X	chr21:36171607	NM_001754	casava
07H099	*KIT*	T417FsX	chr4:55589767	NM_001093772	casava
07H160	*NRAS*	Q61R	chr1:115256529	NM_002524	casava
08H048	*CEBPA*	T191FsX	chr19:33792390	NM_001285829	casava
08H048	*CEBPA*	A30FsX	chr19:33793231	NM_004364	casava
08H048	*KRAS*	A18D	chr12:25398266	NM_033360	kmer
08H048	*WT1*	H246FsX	chr11:32450075	NM_024426	casava
08H112	*IDH2*	R140Q	chr15:90631934	NM_002168	casava
08H112	*KMT2A*	PTD	NA	NA	tophat
08H112	*SRSF2*	P95H	chr17:74732959	NM_003016	casava
08H112	*STAG2*	Q1117X	chrX:123224496	NM_001042749	casava
08H118	*TP53*	Y73C	chr17:7578235	NM_000546	kmer
09H018	*PAK4*	F17V	chr19:39660242	NM_001014835	casava
09H031	*FLT3*	F590FsX, ITD	chr13:28608286	NM_004119	kmer
09H031	*NPM1*	L287FsX	chr5:170837543	NM_002520	casava
09H031	*NPM1*	W288FsX	chr5:170837548	NM_002520	kmer
09H031	*PTPN11*	D61H	chr12:112888165	NM_002834	casava
09H031	*STAG2*	A582FsX	chrX:123196977	NM_001042749	casava
09H046	*BCOR*	R1183X	chrX:39923059	NM_001123383	casava
09H046	*CEBPA*	F60FsX	chr19:33792784	NM_001285829	casava
09H046	*CUX1*	H1183N	chr7:101877412	NM_001202543	casava
09H046	*DNMT3A*	P904L	chr2:25457176	NM_022552	casava
09H046	*DNMT3A*	R899C	chr2:25457192	NM_022552	casava
09H046	*KMT2C*	R2066X	chr7:151878749	NM_170606	casava
09H046	*KMT2D*	R2005C	chr12:49435968	NM_003482	casava
09H046	*NF1*	R1968X	chr17:29661945	NM_001042492	casava
09H046	*RUNX1*	G199W	chr21:36231789	NM_001754	casava
09H046	*SMC5*	R1031Q	chr9:72965314	NM_015110	casava
09H046	*STAG2*	R259X	chrX:123181311	NM_001042749	casava
09H046	*TET2*	R1214Q	chr4:106164773	NM_001127208	casava
09H046	*TET2*	R1452X	chr4:106193892	NM_001127208	casava
09H046	*TP53*	R81X	chr17:7578212	NM_000546	casava
09H046	*USP7*	K695N	chr16:8995054	NM_003470	casava
09H054	*JAK2*	V617F	chr9:5073770	NM_004972	casava
09H113	*DNMT3A*	G890S	chr2:25457219	NM_022552	casava
09H113	*IDH2*	R172K	chr15:90631838	NM_002168	casava
09H113	*KMT2A*	PTD	NA	NA	tophat
09H113	*RUNX1*	RUNX1-SON fusion	NA	NA	manual
10H109	*NPM1*	L258FsX	chr5:170837543	NM_002520	casava
10H109	*NPM1*	W288FsX	chr5:170837548	NM_002520	kmer
10H109	*PTPN11*	A72T	chr12:112888198	NM_002834	kmer
10H109	*TET2*	Q744X	chr4:106157329	NM_017628	casava
10H109	*TET2*	C1289S	chr4:106180837	NM_001127208	casava
11H008	*FLT3*	Y591FsX, ITD	chr13:28608283	NM_004119	kmer
11H008	*KMT2A*	PTD	NA	NA	tophat
11H008	*U2AF1*	S34F	chr21:44524456	NM_001025203	casava
11H129	*GATA2*	L321P	chr3:128202758	NM_001145661	casava
11H129	*NRAS*	G12D	chr1:115258747	NM_002524	casava
11H129	*RAD21*	K605FsX	chr8:117859820	NM_006265	casava
11H151	*DNMT3A*	R736C	chr2:25463287	NM_022552	casava
11H151	*ETV6*	P223L	chr12:12022562	NM_001987	casava
11H151	*FLT3*	D835Y	chr13:28592642	NM_004119	casava
11H151	*NPM1*	L287FsX	chr5:170837543	NM_002520	casava
11H151	*NPM1*	W288FsX	chr5:170837548	NM_002520	kmer
11H151	*TET2*	C1298FsX	chr4:106180864	NM_001127208	casava
12H030	*ASXL1*	E824FsX	chr20:31022985	NM_015338	casava
12H030	*IDH2*	R140Q	chr15:90631934	NM_002168	casava
12H030	*RUNX1*	R204X	chr21:36231774	NM_001754	casava
12H030	*RUNX1*	R166X	chr21:36252866	NM_001754	casava

Supplementary Table 4
Patient mutational data.

CCC	Representative compound	Putative targets	Maximum (RPKM)	Minimum (RPKM)	Median of CCC Resistant specimens (RPKM)	Median of CCC Sensitive specimens (RPKM)	Fold change S/R	FDR q-value
CCC1	Dexamethasone	*NR3C1*	17.4	5.2	8.6	11.8	1.4	n.s.
		NOS2	1.7	0.0	0.0	0.1	5.4	n.s.
		NR0B1	1.8	0.0	0.0	0.0	0.0	n.s.
		ANXA1	2103.0	77.9	868.1	547.7	0.6	n.s.
CCC2	SU5416	*KDR*	0.3	0.0	0.0	0.0	0.0	n.s.
		PDGFRB	5.8	0.0	0.5	0.1	0.3	n.s.
		SRC	16.0	0.5	7.6	6.0	0.8	n.s.
		FGFR1	34.3	0.4	2.4	0.8	0.3	n.s.
		KIT	112.5	0.6	20.5	20.3	1.0	n.s.
		FLT3	333.3	1.7	55.0	75.7	1.4	n.s.
CCC3	Deguelin	*HSP90AA1*	461.4	93.3	173.4	232.4	1.3	n.s.
		HSP90AB1	992.5	156.0	432.2	434.6	1.0	n.s.
		FGFR4	0.8	0.0	0.2	0.2	1.1	n.s.
		NFKBIA	824.3	65.5	250.3	176.8	0.7	n.s.
		XIAP	6.5	1.5	3.3	3.5	1.1	n.s.
		BIRC5	15.9	0.5	2.6	3.8	1.5	n.s.
		MET	0.3	0.0	0.0	0.0	0.6	n.s.
		AKT1	108.3	22.1	60.9	65.0	1.1	n.s.
		AKT2	34.5	16.0	26.1	23.4	0.9	n.s.
		AKT3	11.6	0.0	0.5	0.9	2.0	n.s.
CCC4	Clomiphene Citrate (Z,E)	*ESR1*	0.6	0.0	0.0	0.0	6.4	n.s.
CCC5	Proadifen Hydrochloride	*POR*	24.2	3.9	12.1	11.9	1.0	n.s.
		CYP11A1	8.2	0.0	0.0	0.0	0.3	n.s.
		CYP11B1	0.0	0.0	0.0	0.0	4.5	n.s.
		CYP11B2	0.0	0.0	0.0	0.0	NA	n.s.
		CYP17A1	0.2	0.0	0.0	0.0	4.2	n.s.
		CYP19A1	0.5	0.0	0.0	0.0	0.3	n.s.
		CYP1A1	0.0	0.0	0.0	0.0	0.4	n.s.
		CYP1A2	0.0	0.0	0.0	0.0	NA	n.s.
		CYP1B1	2.6	0.0	0.2	0.1	0.5	n.s.
		CYP20A1	2.6	0.6	1.3	1.2	0.9	n.s.
		CYP21A2	0.2	0.0	0.0	0.0	0.5	n.s.
		CYP24A1	0.0	0.0	0.0	0.0	NA	n.s.
		CYP26A1	0.1	0.0	0.0	0.0	0.7	n.s.
		CYP26B1	0.0	0.0	0.0	0.0	1.4	n.s.
		CYP26C1	0.0	0.0	0.0	0.0	NA	n.s.
		CYP27A1	19.7	0.0	0.8	0.6	0.8	n.s.
		CYP27B1	1.2	0.1	0.5	0.7	1.4	n.s.
		CYP27C1	0.0	0.0	0.0	0.0	0.0	n.s.
		CYP2A13	0.0	0.0	0.0	0.0	NA	n.s.
		CYP2A6	0.3	0.0	0.0	0.1	2.1	n.s.
		CYP2A7	0.0	0.0	0.0	0.0	1.4	n.s.
		CYP2B6	0.0	0.0	0.0	0.0	NA	n.s.
		CYP2C18	0.5	0.0	0.0	0.0	0.9	n.s.
		CYP2C19	0.3	0.0	0.0	0.0	0.0	n.s.
		CYP2C8	2.5	0.0	0.2	0.4	1.7	n.s.
		CYP2C9	0.4	0.0	0.0	0.0	7.5	n.s.
		CYP2D6	0.8	0.0	0.1	0.2	2.7	n.s.
		CYP2E1	6.8	0.0	1.2	1.4	1.2	n.s.
		CYP2F1	0.6	0.0	0.1	0.1	1.6	n.s.
		CYP2J2	0.6	0.0	0.0	0.0	1.2	n.s.
		CYP2R1	8.3	0.8	4.2	4.7	1.1	n.s.
		CYP2S1	11.4	0.1	1.9	3.7	2.0	n.s.
		CYP2U1	2.0	0.0	0.6	0.3	0.6	n.s.
		CYP2W1	0.1	0.0	0.0	0.0	1.3	n.s.
		CYP39A1	0.0	0.0	0.0	0.0	NA	n.s.
		CYP3A43	0.1	0.0	0.0	0.0	0.6	n.s.
		CYP3A4	0.1	0.0	0.0	0.0	0.5	n.s.
		CYP3A5	0.6	0.0	0.1	0.1	1.4	n.s.
		CYP3A7	0.1	0.0	0.0	0.0	NA	n.s.
		CYP46A1	2.9	0.0	0.0	0.0	1.1	n.s.
		CYP4A11	0.0	0.0	0.0	0.0	NA	n.s.
		CYP4A22	0.0	0.0	0.0	0.0	NA	n.s.
		CYP4B1	0.0	0.0	0.0	0.0	NA	n.s.
		CYP4F11	1.1	0.0	0.0	0.0	0.7	n.s.
		CYP4F12	1.7	0.0	0.0	0.0	0.7	n.s.
		CYP4F22	1.3	0.0	0.0	0.0	2.3	n.s.
		KCNJ8	0.2	0.0	0.0	0.0	NA	n.s.
		NOS1	0.2	0.0	0.0	0.0	NA	n.s.

Supplementary Table 5
Non-exhaustive list of putative targets from Compound Correlation Clusters (CCCs) representative compounds and their expression levels in patient specimens.

Compound A name	Compound B name	Patient number	Synergistic area	Antagonistic area	Deviation from additivity	Cummulative synergy effect	Ratio
Dexamethasone	SU 5416	04H112	0	53725	53725	-53725	NA
Dexamethasone	Deguelin	04H112	167	1883	2050	-1716	-1.1
Dexamethasone	Clomiphene citrate	04H112	0	3442	3442	-3442	NA
Dexamethasone	Proadifen hydrochloride	04H112	249	5275	5524	-5026	-1.3
Dexamethasone	Mubritinib	04H112	0	1966	1966	-1966	NA
SU 5416	Deguelin	04H112	2321	2543	4864	-222	0.0
SU 5416	Clomiphene citrate	04H112	0	10369	10369	-10369	NA
SU 5416	Proadifen hydrochloride	04H112	438	7096	7534	-6658	-1.2
SU 5416	Mubritinib	04H112	21	3294	3315	-3273	-2.2
Deguelin	Clomiphene citrate	04H112	1518	0	1518	1518	3.0
Deguelin	Proadifen hydrochloride	04H112	3311	0	3311	3311	3.0
Clomiphene citrate	Proadifen hydrochloride	04H112	4431	42	4473	4389	2.0
Clomiphene citrate	Mubritinib	04H112	434	0	434	434	3.0
Proadifen hydrochloride	Mubritinib	04H112	1210	38	1248	1172	1.5
Dexamethasone	SU 5416	06H133	0	33219	33219	-33219	NA
Dexamethasone	Deguelin	06H133	20177	43	20220	20134	2.7
Dexamethasone	Clomiphene citrate	06H133	18212	1	18213	18211	3.0
Dexamethasone	Proadifen hydrochloride	06H133	37183	24	37207	37159	3.0
Dexamethasone	Mubritinib	06H133	5483	602	6085	4881	1.0
SU 5416	Deguelin	06H133	382	131	513	251	0.5
SU 5416	Clomiphene citrate	06H133	4763	0	4763	4763	3.0
SU 5416	Proadifen hydrochloride	06H133	10341	0	10341	10341	3.0
SU 5416	Mubritinib	06H133	9	848	857	-839	-2.0
Deguelin	Clomiphene citrate	06H133	3072	2	3074	3070	3.0
Deguelin	Proadifen hydrochloride	06H133	6057	0	6057	6057	3.0
Clomiphene citrate	Proadifen hydrochloride	06H133	5819	0	5819	5819	3.0
Clomiphene citrate	Mubritinib	06H133	666	313	979	353	0.3
Proadifen hydrochloride	Mubritinib	06H133	2347	520	2867	1827	0.7
Dexamethasone	SU 5416	07H069	0	1324	1324	-1324	NA
Dexamethasone	Deguelin	07H069	277	391	668	-114	-0.1
Dexamethasone	Clomiphene citrate	07H069	0	1397	1397	-1397	NA
Dexamethasone	Proadifen hydrochloride	07H069	35	3322	3357	-3287	-2.0
Dexamethasone	Mubritinib	07H069	402	435	837	-33	0.0
SU 5416	Deguelin	07H069	10128	28	10156	10100	2.6
SU 5416	Clomiphene citrate	07H069	28909	1	28910	28908	3.0
SU 5416	Proadifen hydrochloride	07H069	655	6217	6872	-5562	-1.0
SU 5416	Mubritinib	07H069	1860	225	2085	1635	0.9
Deguelin	Clomiphene citrate	07H069	34392	0	34392	34392	3.0
Deguelin	Proadifen hydrochloride	07H069	11772	8	11780	11764	3.0
Clomiphene citrate	Proadifen hydrochloride	07H069	6554	0	6554	6554	3.0
Clomiphene citrate	Mubritinib	07H069	38384	23	38407	38361	3.0
Proadifen hydrochloride	Mubritinib	07H069	17453	7	17460	17446	3.0
Dexamethasone	SU 5416	09H031	8287	1560	9847	6727	0.7
Dexamethasone	Deguelin	09H031	20611	463	21074	20148	1.6
Dexamethasone	Clomiphene citrate	09H031	20298	0	20298	20298	3.0
Dexamethasone	Proadifen hydrochloride	09H031	5225	2173	7398	3052	0.4
Dexamethasone	Mubritinib	09H031	1198	5782	6980	-4584	-0.7
SU 5416	Deguelin	09H031	0	16432	16432	-16432	NA
SU 5416	Clomiphene citrate	09H031	3385	201	3586	3184	1.2
SU 5416	Proadifen hydrochloride	09H031	93	7285	7378	-7192	-1.9
SU 5416	Mubritinib	09H031	0	27183	27183	-27183	NA
Deguelin	Clomiphene citrate	09H031	31035	0	31035	31035	3.0
Deguelin	Proadifen hydrochloride	09H031	6496	547	7043	5949	1.1
Clomiphene citrate	Proadifen hydrochloride	09H031	14476	0	14476	14476	3.0
Clomiphene citrate	Mubritinib	09H031	5475	441	5916	5034	1.1
Proadifen hydrochloride	Mubritinib	09H031	27119	0	27119	27119	3.0

Supplementary Table 6 (1/3)

Raw data of synergistic screen.

Compound A name	Compound B name	Patient number	Synergistic area	Antagonistic area	Deviation from additivity	Cummulative synergy effect	Ratio
Dexamethasone	SU 5416	09H111	70	8133	8203	-8063	-2.1
Dexamethasone	Deguelin	09H111	21111	2	21113	21109	3.0
Dexamethasone	Clomiphene citrate	09H111	25560	0	25560	25560	3.0
Dexamethasone	Proadifen hydrochloride	09H111	6350	803	7153	5547	0.9
Dexamethasone	Mubritinib	09H111	13171	38	13209	13133	2.5
SU 5416	Deguelin	09H111	0	18754	18754	-18754	NA
SU 5416	Clomiphene citrate	09H111	0	6502	6502	-6502	NA
SU 5416	Proadifen hydrochloride	09H111	1078	4333	5411	-3255	-0.6
SU 5416	Mubritinib	09H111	0	18341	18341	-18341	NA
Deguelin	Clomiphene citrate	09H111	96	20732	20828	-20636	-2.3
Deguelin	Proadifen hydrochloride	09H111	11419	1723	13142	9696	0.8
Clomiphene citrate	Proadifen hydrochloride	09H111	15842	0	15842	15842	3.0
Clomiphene citrate	Mubritinib	09H111	3677	3263	6940	414	0.1
Proadifen hydrochloride	Mubritinib	09H111	532	13325	13857	-12793	-1.4
Dexamethasone	SU 5416	09H113	0	5946	5946	-5946	NA
Dexamethasone	Deguelin	09H113	1890	0	1890	1890	3.0
Dexamethasone	Clomiphene citrate	09H113	2130	276	2406	1854	0.9
Dexamethasone	Proadifen hydrochloride	09H113	1131	1129	2260	2	0.0
Dexamethasone	Mubritinib	09H113	4	969	973	-965	-2.4
SU 5416	Deguelin	09H113	1156	775	1931	381	0.2
SU 5416	Clomiphene citrate	09H113	1430	692	2122	738	0.3
SU 5416	Proadifen hydrochloride	09H113	51	4068	4119	-4017	-1.9
SU 5416	Mubritinib	09H113	12	3832	3844	-3820	-2.5
Deguelin	Clomiphene citrate	09H113	20605	0	20605	20605	3.0
Deguelin	Proadifen hydrochloride	09H113	9500	200	9700	9300	1.7
Clomiphene citrate	Proadifen hydrochloride	09H113	2519	653	3172	1866	0.6
Clomiphene citrate	Mubritinib	09H113	11263	0	11263	11263	3.0
Proadifen hydrochloride	Mubritinib	09H113	7085	38	7123	7047	2.3
Dexamethasone	SU 5416	10H101	2611	9272	11883	-6661	-0.6
Dexamethasone	Deguelin	10H101	2849	4647	7496	-1798	-0.2
Dexamethasone	Clomiphene citrate	10H101	0	24764	24764	-24764	NA
Dexamethasone	Proadifen hydrochloride	10H101	0	17480	17480	-17480	NA
Dexamethasone	Mubritinib	10H101	829	5563	6392	-4734	-0.8
SU 5416	Deguelin	10H101	32	4973	5005	-4941	-2.2
SU 5416	Clomiphene citrate	10H101	1683	4212	5895	-2529	-0.4
SU 5416	Proadifen hydrochloride	10H101	2099	1969	4068	130	0.0
SU 5416	Mubritinib	10H101	0	5809	5809	-5809	NA
Deguelin	Clomiphene citrate	10H101	12094	0	12094	12094	3.0
Deguelin	Proadifen hydrochloride	10H101	9558	0	9558	9558	3.0
Clomiphene citrate	Proadifen hydrochloride	10H101	19557	0	19557	19557	3.0
Clomiphene citrate	Mubritinib	10H101	3823	0	3823	3823	3.0
Proadifen hydrochloride	Mubritinib	10H101	6099	0	6099	6099	3.0

Supplementary Table 6 (2/3)
Raw data of synergistic screen.

Compound A name	Compound B name	Patient number	Synergistic area	Antagonistic area	Deviation from additivity	Cummulative synergy effect	Ratio
Dexamethasone	SU 5416	10H166	10791	6637	17428	4154	0.2
Dexamethasone	Deguelin	10H166	14773	144	14917	14629	2.0
Dexamethasone	Clomiphene citrate	10H166	5	14651	14656	-14646	-3.5
Dexamethasone	Proadifen hydrochloride	10H166	3	17818	17821	-17815	-3.8
Dexamethasone	Mubritinib	10H166	3971	238	4209	3733	1.2
SU 5416	Deguelin	10H166	43	7361	7404	-7318	-2.2
SU 5416	Clomiphene citrate	10H166	233	8328	8561	-8095	-1.6
SU 5416	Proadifen hydrochloride	10H166	1359	4376	5735	-3017	-0.5
SU 5416	Mubritinib	10H166	68	4019	4087	-3951	-1.8
Deguelin	Clomiphene citrate	10H166	14402	1	14403	14401	3.0
Deguelin	Proadifen hydrochloride	10H166	8998	0	8998	8998	3.0
Clomiphene citrate	Proadifen hydrochloride	10H166	5575	0	5575	5575	3.0
Clomiphene citrate	Mubritinib	10H166	7852	0	7852	7852	3.0
Proadifen hydrochloride	Mubritinib	10H166	8231	97	8328	8134	1.9
Dexamethasone	SU 5416	12H030	0	1217	1217	-1217	NA
Dexamethasone	Deguelin	12H030	873	1116	1989	-243	-0.1
Dexamethasone	Clomiphene citrate	12H030	50	1435	1485	-1385	-1.5
Dexamethasone	Proadifen hydrochloride	12H030	431	1249	1680	-818	-0.5
Dexamethasone	Mubritinib	12H030	40	1373	1413	-1333	-1.5
SU 5416	Deguelin	12H030	1	3253	3254	-3252	-3.5
SU 5416	Clomiphene citrate	12H030	22	1209	1231	-1187	-1.7
SU 5416	Proadifen hydrochloride	12H030	0	1270	1270	-1270	NA
SU 5416	Mubritinib	12H030	0	1969	1969	-1969	NA
Deguelin	Clomiphene citrate	12H030	13675	4	13679	13671	3.0
Deguelin	Proadifen hydrochloride	12H030	19124	0	19124	19124	3.0
Clomiphene citrate	Proadifen hydrochloride	12H030	11595	0	11595	11595	3.0
Clomiphene citrate	Mubritinib	12H030	16201	0	16201	16201	3.0
Proadifen hydrochloride	Mubritinib	12H030	33966	4	33970	33962	3.0
Dexamethasone	SU 5416	mPB1	23	13873	13896	-13850	-2.8
Dexamethasone	Deguelin	mPB1	26	1080	1106	-1054	-1.6
Dexamethasone	Clomiphene citrate	mPB1	3000	1042	4042	1958	0.5
Dexamethasone	Proadifen hydrochloride	mPB1	4129	2349	6478	1780	0.2
Dexamethasone	Mubritinib	mPB1	0	2298	2298	-2298	NA
SU 5416	Deguelin	mPB1	681	1508	2189	-827	-0.3
SU 5416	Clomiphene citrate	mPB1	3990	445	4435	3545	1.0
SU 5416	Proadifen hydrochloride	mPB1	13641	217	13858	13424	1.8
SU 5416	Mubritinib	mPB1	123	1681	1804	-1558	-1.1
Deguelin	Clomiphene citrate	mPB1	1588	835	2423	753	0.3
Deguelin	Proadifen hydrochloride	mPB1	2122	1252	3374	870	0.2
Clomiphene citrate	Proadifen hydrochloride	mPB1	4429	0	4429	4429	3.0
Clomiphene citrate	Mubritinib	mPB1	781	1193	1974	-412	-0.2
Proadifen hydrochloride	Mubritinib	mPB1	635	2250	2885	-1615	-0.5
Dexamethasone	SU 5416	mPB2	0	30863	30863	-30863	NA
Dexamethasone	Deguelin	mPB2	357	765	1122	-408	-0.3
Dexamethasone	Clomiphene citrate	mPB2	2847	2116	4963	731	0.1
Dexamethasone	Proadifen hydrochloride	mPB2	2965	2383	5348	582	0.1
Dexamethasone	Mubritinib	mPB2	1	1894	1895	-1893	-3.3
SU 5416	Deguelin	mPB2	539	2710	3249	-2171	-0.7
SU 5416	Clomiphene citrate	mPB2	4286	35	4321	4251	2.1
SU 5416	Proadifen hydrochloride	mPB2	7426	355	7781	7071	1.3
SU 5416	Mubritinib	mPB2	0	1675	1675	-1675	NA
Deguelin	Clomiphene citrate	mPB2	6987	70	7057	6917	2.0
Deguelin	Proadifen hydrochloride	mPB2	3216	681	3897	2535	0.7
Clomiphene citrate	Proadifen hydrochloride	mPB2	1137	2211	3348	-1074	-0.3
Clomiphene citrate	Mubritinib	mPB2	1446	961	2407	485	0.2
Proadifen hydrochloride	Mubritinib	mPB2	584	1560	2144	-976	-0.4

Supplementary Table 6 (3/3)
Raw data of synergistic screen.
